# Taxonomic Characterization of Honey Bee (*Apis mellifera*) Pollen Foraging Based on Non-Overlapping Paired-End Sequencing of Nuclear Ribosomal Loci

**DOI:** 10.1371/journal.pone.0145365

**Published:** 2015-12-23

**Authors:** R. Scott Cornman, Clint R. V. Otto, Deborah Iwanowicz, Jeffery S. Pettis

**Affiliations:** 1 U.S. Geological Survey, Fort Collins Science Center, 2150 Centre Avenue, Fort Collins, CO, 80526, United States of America; 2 U.S. Geological Survey, Northern Prairie Wildlife Research Center, 8711 37th Street Southeast, Jamestown, ND, 58401, United States of America; 3 U.S. Geological Survey, Leetown Science Center, 11649 Leetown Road, Kearneysville, WV, 25430, United States of America; 4 U.S. Department of Agriculture-Agriculture Research Service, Beltsville Agricultural Research Center, Bee Research Laboratory, 10300 Baltimore Avenue, Beltsville, MD, 20705, United States of America; University of British Columbia, CANADA

## Abstract

Identifying plant taxa that honey bees (*Apis mellifera*) forage upon is of great apicultural interest, but traditional methods are labor intensive and may lack resolution. Here we evaluate a high-throughput genetic barcoding approach to characterize trap-collected pollen from multiple North Dakota apiaries across multiple years. We used the Illumina MiSeq platform to generate sequence scaffolds from non-overlapping 300-bp paired-end sequencing reads of the ribosomal internal transcribed spacers (ITS). Full-length sequence scaffolds represented ~530 bp of ITS sequence after adapter trimming, drawn from the 5’ of ITS1 and the 3’ of ITS2, while skipping the uninformative 5.8S region. Operational taxonomic units (OTUs) were picked from scaffolds clustered at 97% identity, searched by BLAST against the nt database, and given taxonomic assignments using the paired-read lowest common ancestor approach. Taxonomic assignments and quantitative patterns were consistent with known plant distributions, phenology, and observational reports of pollen foraging, but revealed an unexpected contribution from non-crop graminoids and wetland plants. The mean number of plant species assignments per sample was 23.0 (+/- 5.5) and the mean species diversity (effective number of equally abundant species) was 3.3 (+/- 1.2). Bray-Curtis similarities showed good agreement among samples from the same apiary and sampling date. Rarefaction plots indicated that fewer than 50,000 reads are typically needed to characterize pollen samples of this complexity. Our results show that a pre-compiled, curated reference database is not essential for genus-level assignments, but species-level assignments are hindered by database gaps, reference length variation, and probable errors in the taxonomic assignment, requiring post-hoc evaluation. Although the effective per-sample yield achieved using custom MiSeq amplicon primers was less than the machine maximum, primarily due to lower “read2” quality, further protocol optimization and/or a modest reduction in multiplex scale should offset this difficulty. As small quantities of pollen are sufficient for amplification, our approach might be extendable to other questions or species for which large pollen samples are not available.

## Introduction

Understanding mutualistic interactions between plants and animal pollinators is important for sustaining ecosystem function and maintaining pollinator communities that support agriculture [1–2]. In the U.S., the economic value of crop pollination by native and non-native insects, most prominently the honey bee (*Apis mellifera*), is estimated at $15 billion (USD) annually [3]. Reliance on insects for pollination services is growing even as populations of native and domestic pollinators are in decline [4–5]. Factors attributed to pollinator declines include reduced forage diversity and abundance, land-use change, pathogens, disease, pesticide exposure, and socio-economic factors [6–8]. Widespread decline in native and non-native insects puts considerable pressure on agricultural productivity and global food supplies [9]. As a result, U.S. pollinator conservation has recently become a national issue. In May of 2015 a US federal strategy was developed to promote the health of honey bees and other pollinators [10]. One of the key objectives of the federal strategy includes the establishment of 7 million acres of pollinator habitat by 2020.

In light of recent pollinator declines and potential loss of pollination services, the scientific community has called for a redoubling of applied research of pollinator systems [11]. In particular, improving resolution of pollinator forage is of great practical value, such as in the design of seeding mixes for specific conservation goals. Techniques for assessing plant-pollinator interactions in such contexts include direct observation of insect visitation to individual flowers (e.g., [12–14]), mark-recapture (e.g., [15]), agent-based foraging models (e.g., [16]), digital tracking devices (e.g., [17]), chemical signatures (e.g., [18]), genetic sequencing (e.g., [19–23]) and light microscopy (e.g., [15,24,25]). Among these, techniques that identify pollen collected from insect bodies or nests may provide a more holistic understanding of plant-pollinator interactions than single observations of insect-flower visits [24,25]. These pollen mixtures have most often been analyzed via light microscopy using dichotomous keys, a process that is laborious, requires expertise in pollen identification, and typically identifies only a subset of the taxonomic diversity.

Sequencing of taxonomically informative “barcode” genetic loci has emerged over the past two decades as a robust tool for taxonomic classification. More recently, high-throughput sequencing has allowed “meta-barcoding” analysis of diverse mixtures, and this approach has begun to be applied to bee-collected pollen [20–23]. In principle, taxonomic profiles can now be quickly generated for dozens of milligram-scale pollen samples in a single sequencing run without specialized training in the visual classification of pollen. These techniques promise an important advance in understanding plant-pollinator interactions and the foraging needs of pollinating insects, but require validation so that the results can be interpreted with confidence.

Here we investigate a metagenetic classification of pollen forage collected by honey bees in agricultural and uncultivated grassland landscapes of North Dakota, a major summering ground for commercial hives. Our sequencing approach differs from those recently published in one or more ways: the sequence that was analyzed, the use of a higher-capacity platform with more flexible multiplexing, or the development of a workflow for non-overlapping read pairs. We evaluate the concordance of taxon assignments with available phenological and distributional records, and assess the robustness of taxonomic recovery with rarefaction and replication analysis. Our results provide an initial picture of temporal and landscape patterns of foraging behavior needed to guide pollen sampling effort, and to evaluate barcode-sequence gaps specific to the region, an area of great relevance to U.S. apiculture.

## Methods

### Study Area

We conducted our study at six commercial apiaries (*i*.*e*., sites) in the “Prairie Pothole” Region of southeastern North Dakota, U.S. (S1 File). These apiaries have been part of an ongoing study of honey bee forage and landscape health since 2009 [26]. Apiaries were selected to reflect a spectrum of land cover and use, such as pasture, hay fields, cover crops, soybeans, small grains, corn, other mix crops, and isolated wetlands. Browning’s Honey Company provided the research colonies and permission to access them, and assisted with their management. Field studies did not involve endangered or protected species. No animals were euthanized in this study. Because honey bees are invertebrates, the study did not fall under the purview of our Institutional Animal Care and Use Committee.

### Background on Pollen Foraging in Honey Bees

Honey bees collect pollen because it provides the colony with amino acids, vitamins, minerals, and lipids needed for brood production [27]. Nutritional quality of pollen has been linked with honey bee physiology, worker longevity, and tolerance of parasitism [28,29]. Pollen stores within the hive are utilized extensively by nurse bees for rearing brood during the spring, summer, and fall. Pollen collection and storage levels may be important fitness metrics because they are related to bee physiology, colony brood production, and future colony size [30]. A typical honey bee colony will collect 13–20 kg of pollen each year and store ≤ 1 kg of pollen within the hive [31,32]. Pollen collection rates are regulated within the hive based on the quantity of stored pollen and pollen consumption rates [30,33]. Emission of brood pheromone by larval bees may also stimulate pollen foraging [34]. About 130 mg of pollen is required for rearing a single bee, and a colony may raise over 100,000 bees during the summer [35]. Although individual honey bees maintain high species fidelity during specific foraging trips, a bee colony as a whole can collect pollen from a variety of plant species throughout a day. While foraging, bees mix pollen with regurgitated nectar or ‘glue’ honey [36] and other secretions, which is then groomed from the body and packed into a specialized storage structure on each of the rear legs (the corbicula or “pollen baskets”). It is reasonable to suspect that some amount of passively acquired pollen (i.e., not from plants visited by the forager but incidentally encountered through carryover from other pollinators, wind transport, physical contact between flowers of different species, etc.) is incorporated into these pollen loads, but this study was not designed to distinguish such.

### Pollen Sample Collection

We used pollen traps to collect pollen samples from three randomly selected honey bee colonies within each apiary, collected as part of the larger research program mentioned above. CC Pollen Traps (Dadant and Sons, Inc.) were positioned at the base of each honey bee colony and activated for approximately 72 hours, every 2–3 weeks. The same colonies were sampled throughout each season. When open, the pollen trap forces foragers to return through a series of 5-mm x 5-mm mesh screens before they can enter the main chamber of the hive, stripping pollen loads from the corbicula into a collection pan. Traps are activated by sealing the main entrance into the hive and opening the entrance into the pollen trap. Traps are deactivated using the inverse procedure. For each trapping event a random 15-g grab sample was collected. Pollen samples were pre-processed to remove detritus such as dead bees and wax prior to storage at -20°C.

For this methods development, we used a subsample of pollen archived from the summers of 2009 and 2010. Samples were selected to represent a breadth of the foraging season as well as variation across sites and years, constrained by an initial multiplexing goal of 96 samples. Furthermore, not all colonies collected enough pollen needed for sample archiving at each trapping event. As a result, representation and replication by year and site varies due to our exploratory approach and natural variation in honey bee pollen collection.

### Sequencing strategy

We developed a sequencing strategy for the Illumina MiSeq platform because it then offered the most suitable combination of read pairing, amplicon-end sequencing, read length, read quality, and per-sample cost. Nonetheless, a significant drawback of the standard MiSeq full-length amplicon protocol (http://support.illumina.com/documents/documentation/chemistry_documentation/16s/16s-metagenomic-library-prep-guide-15044223-b.pdf) is the need for long custom primers of ~70 bp that fuse sequencing adapters to the barcode primer, and which must amplify at 60–65°C. We chose to target the internal transcribed spacer (ITS) of the nuclear ribosomal locus [37] for protocol development, in part because of concerns that chloroplast loci would not be reliably or proportionally amplified from all pollen types (chloroplasts are excluded from the pollen gametes during pollen development but may be present in other pollen tissue to an extent that varies by species [38,39]. We were also concerned that the generally lower GC content of chloroplast relative to nuclear sequence could contribute to greater taxonomic dropout at chloroplast loci under high annealing temperatures. Indeed, an initial trial of adapted trnH-psbA primers [37] amplified poorly in our hands, although [22] have since demonstrated excellent performance of the chloroplast trnL locus for pollen metabarcoding. We therefore developed custom primers extending the published “ITS4” [40] and “ITS5a” [41] combination extended with Illumina sequence adapters (underlined): GTC TCG TGG GCT CGG AGA TGT GTA TAA GAG ACA GTT CCT CCG CTT ATT GAT ATG CTT AAR YTC AGC (modified ITS4) and TCG TCG GCA GCG TCA GAT GTG TAT AAG AGA CAG ACC TTA TCA TTT AGA GGA AGK ARA ART CGT AAC AAG GT (modified ITS5a). These primers produced a ~900-bp fragment that could be subjected to 300-bp paired-end sequencing, recovering informative ITS sequence from both spacer regions (ITS1 and ITS2) without redundancy, while avoiding the conserved, less informative 5.8S region in the middle of the amplicon [42].

### DNA Extraction

Each sample was homogenized using a mortar and pestle and dried in an oven at 60°C for 60 hours. Dried pollen samples were shipped to the U.S. Geological Survey (USGS), Leetown Science Center, Kearneysville, WV, for meta-genetic analysis. Samples were held at room temperature until DNA extraction using a modification of Doyle’s method [43]. 600 μL of 55°C cetyltrimethyl ammonium bromide (CTAB) lysis buffer (PanReac Applichem) was added to 25–40 mg of pollen in a 2-mL round-bottom microcentrifuge tube loaded with a 3-mm stainless steel disruption bead. Tubes were shaken on a Qiagen TissueLyser II for 4 minutes at 30 hz and then incubated at 55°C for 30 minutes with occasional flicks to ensure adequate mixing. Afterward, 500 μL of a 24:1 chloroform:isoamyl alcohol solution was added. After 30 s of gentle inversions, the sample was briefly vortexed and centrifuged at 3,300 g for 10 min. The top phase was transferred to a new microcentrifuge tube and a second aliquot (1/5 volume) of 55°C CTAB lysis buffer was added. After a 15-min incubation at 55°C, a second chloroform:isoamyl alcohol extraction was performed, followed by a 10-min centrifugation at 8500 rpm (6900 g). The top phase was transferred to a new tube, and a 1/10 volume of 1 M ammonium acetate and 0.7 volume of ice-cold isopropanol were added. Tubes were then incubated at 4°C for 1 hr and then centrifuged at 13,400 g for 15 min. Isopropanol was poured off and 500 μL of 70% ethanol was added. After gentle mixing, the tube was centrifuged at 3,300 g for 60 seconds. The ethanol was poured off and the pellet was allowed to air dry under a ventilation hood. Finally, the pellet was rehydrated in 50 μL of nuclease free water and held at 4°C for up to 12 hours before further processing.

### PCR Amplification

ITS regions were initially amplified with the standard ITS5a and ITS4 primers [40] at a 1-μM concentration. The amplification reaction contained 0.15 μM of each primer, 1 μL of the initial amplification product, and Promega GoTaq Green Master Mix following manufacturer recommendations for a 25-μL reaction. The thermocycler program consisted of an initial denaturation step of 95°C for 5 min, followed by 40 cycles of 30 s at 95°C, 35 s at 47°C, and 1.5 min at 72°C. Products were subjected to a final extension at 72°C for 10 minutes and then held until collection at 4°C. An appropriately sized amplification product was confirmed for each reaction by electrophoresis of 5 μL of the reaction product through a 1.5% I.D.NA agarose gel (FMC Bioproducts) at 100 V for 45 min. PCR products were cleaned with the Qiagen Qiaquick PCR purification kit and quantified using the Qubit dsDNA HS Assay Kit (Life Technologies). Samples were diluted in 10 mM Tris buffer (pH 8.5) to a final concentration of 5 ng/μL.

### Library Preparation, Sequencing and Quality Assessment

Multiplex library preparation followed the manufacturer’s protocol for amplicon sequencing. Libraries were diluted 1:10 with nuclease-free water and quantified with the Qubit dsDNA HS Assay Kit. DNA size spectra were determined using an Agilent DNA 1000 kit. After pooling equal concentrations of each sample library, the combined library pool was diluted to 4 nM using 10 mM Tris pH 8.5. A final 15 pM preparation was created with a 6.5% PhiX control spike following the manufacturer’s protocol, which was then sequenced using the MiSeq 2 x 300 bp paired-end run protocol. Machine processed sequencing output has been deposited in GenBank under BioProject PRJNA295334.

### Bioinformatic Analysis

Raw reads were imported into CLC Genomics Workbench v. 7 (Qiagen) for adapter screening and quality trimming. We imposed a maximum error probability of 0.05 for individual bases and a total score of 10 (+1/-2 for matches/mismatches and indels) to trim the forward and reverse fusion primers. Degenerate positions in the primers were accommodated by providing multiple explicit sequence variants as search models. The required minimum read length was 150 bp and at most three ambiguous positions were allowed. Only intact pairs were retained after trimming, and any that overlapped based on the CLC Genomics ‘merge reads’ function were discarded as improper fragments (minimum merge score of 25 with a +1/-2/-3 scoring scheme for matches, mismatches, and indels, respectively). Quality-checked reads were converted to FASTA format for further use.

To evaluate the ability of our approach to identify taxa without using prior knowledge of plant biogeography, phenology, or bee foraging behavior, we used *de novo* operational taxonomic unit (OTU) identification followed by taxonomic assignment with BLAST and the NCBI Taxonomy resource. This strategy was chosen because while considerable prior knowledge exists it is incomplete and not all plausible species are represented by complete ribosomal ITS sequences in GenBank (see below). Nonetheless, we do evaluate our results for consistency with distribution and phenology data from North Dakota floras [44–46], as well as consistency of samples by date and site using similarity scores.

Selection of *de novo* OTUs involves clustering sequence reads into sufficiently similar sets such that they can all be represented by a single member, which is then used as input for downstream processes under the assumption that important taxonomic information is not lost by this grouping. Rather than performing a computationally expensive clustering of all reads together, we used a divide-and-conquer strategy to reduce the total number of pairwise alignments made, whereby reads were binned based on an initial mapping to an ITS reference database drawn from GenBank accessions. The custom database included all entries from the GenBank nucleotide database assigned to the “Angiosperm” taxonomic node that contained “transcribed spacer” or “ITS” in the header, further clustered at 95%. Read pairs were mapped to this database using bowtie2 [47] with the “very-sensitive” and “end-to-end” quick switches and a maximum insert size set to 1100 bp. The resulting alignment file was parsed to seed initial clusters and was not used for any subsequent analysis. At this stage, unmapped reads were not used in OTU generation but were recaptured in a later step, see below.

Because clustering programs typically include a length sorting step and select the longest representative sequence in a cluster, retaining short sequences in each bin is an unnecessary computational cost assuming that all sampled taxa will be represented by at least one high-quality read pair. We therefore only included read pairs in the clustering step that had a combined length ≥ 520 bp after trimming. To format read pairs for clustering, we created scaffolds by reverse-complementing the second read and joining it with the first, with a linking sequence of 30 “N”. (This length of inserted sequence prevented single reads from erroneously mapping across the scaffold gap). We then ran CD-HIT-est [48] on each scaffold pool sequentially with a wrapper script using a 97% identity threshold and otherwise default parameters. While a 97% threshold is used throughout the workflow (see below), this value was chosen operationally and is not an assumption of species-level divergence for these ITS sequences. As evolutionary rates vary, no single value is likely to recapitulate taxonomy, and indeed in our results most species are represented by multiple OTUs but some closely related species cannot be distinguished at this threshold (see below).

A second mapping was performed with bowtie2 of all passed read pairs against the selected cluster representatives. The resulting alignment file was parsed to identify read pairs not mapping to an existing cluster representative at the 97% level; these were re-captured and clustered as a single pool of scaffolds using the CD-HIT-est parameters described above. A third mapping was performed in order to generate a consensus sequence for the final set of OTU clusters, using SAMtools [49] and only those read pairs that mapped at 97% identity with at most five alignment positions represented by indels. Final OTUs were checked for chimeras with UCHIME [50] using the GenBank-sourced ITS database described above as a reference. The scoring parameters were beta = 5, minimum score = 1, and minimum divergence ratio = 1. The choice of a threshold score for chimera removal depends on the properties of the data and reference set, with high-confidence chimeras appearing as strong outliers in the score distribution. Inspection of the data led us to select a relatively high threshold score of 30 for removing chimeric scaffolds, as lower scoring candidates were often proposed chimeras of closely related taxa or were ambiguous when analyzed with BLAST. However, our paired-read assignment criteria (see below and Results) constituted a much stronger filter of potential chimeric sequences, as read pairs with conflicting taxonomic matches are deferred to the next most inclusive taxonomic level or left unassigned (see below).

We observed that G homopolymers were sometimes present at the 3’ end of scaffolds, which we presume to be sequencing artifacts. To prevent these regions from seeding alignments, scaffolds were screened for low-complexity sequence using mdust [51] with default scoring parameters.

We used the metagenomics package Megan v. 5.10.2 [52] extensively for preliminary taxonomic assignments and to analyze read counts, however we did not use the paired-end mode of Megan for final OTU assignment. This was because only a small fraction of Megan assignments actually used the paired BLAST scores from both mates, for undetermined reasons. Instead, we implemented the lowest common ancestor (LCA) approach directly using BLASTN and the NCBI taxonomy resource. To apply this approach to paired reads, we split OTU scaffolds into their component reads and searched the nt database with BLASTN (using default settings for the ‘discontinuous megablast’ option). We then used perl scripts to parse the top 25 matched accessions from the BLAST output, generating a matrix of high-scoring pairs (HSPs) accessions, bit scores, and percent identities. We used the NCBI Taxonomy text dumps (ftp://ftp.ncbi.nlm.nih.gov/pub/taxonomy/) to add the taxonomic classification for each matched accession. To apply the ‘naïve’ LCA algorithm to each OTU, at each of five specified ranks (species, genus, tribe, family, and order) we summed the best score of each read in a pair for each taxon associated with these top 25 accessions. If only one taxon had a combined score > 600 and was within 3% of the best score, the OTU was assigned to that taxon, otherwise the OTU was considered undetermined at that taxonomic level. A 3% cutoff corresponds to a minimum bit-score difference of 18 and an average difference greater than 20 (see Results). We believe a larger percentage cutoff would be overly conservative given the already stringent requirement of concordant 97% mapping of two reads of at least 150 bp each, however, it should be noted that neither OTU assignments nor read mappings are associated with an explicit error probability. We calculated summary statistics for OTU assignments, including mean percent identity and bit scores of HSPs, as well as whether assignments fell within the target group (the green plants, or kingdom Viridiplantae) or were non-target taxa.

A fourth and final mapping generated counts for taxa by sample, summing across OTUs with the same taxon assignment. Again, only pairs that mapped to the same OTU at 97% and 5 or fewer indel positions were counted. We chose to consider invalid read pairs that mapped discordantly across different OTUs even if given the same taxonomic assignment. Their inclusion would have increased the total mapped reads by less than 5%, at a cost of greater uncertainty (OTU assignments vary in strength) and a more burdensome workflow for retrospective reassignment as new reference sequences become available. Read counts are reported as counts per million (cpm) to account for variations in library size.

Read-mapping is an approximate process that results in a nonzero level of suboptimal assignments [53]. For this reason as well as simple heteroscedasticity, low counts are not expected to be robust. As even genuine low-abundance taxa lack biological relevance for our research objectives, we excluded taxa with less than a total of 50 counts summed across all samples. This threshold was chosen subjectively based on the long tail of low-count taxa in the total distribution. Threshold criteria have been included as options in other metabarcoding workflows (e.g., [54,55]) as a standard noise-reduction measure.

Taxonomic diversity of read counts was assessed using the bcDiversity function in the *entropart* R package [56] with ChaoShen bias correction and exponent q = 1. As the diversity value is bias-corrected based on actual sample sizes, raw values rather than library-size normalized cpm were used. Rarefaction curves were obtained with the ‘rarefy’ function of the *vegan* R package [57]. Two samples, A091 and A092 (see S1 File), were suspected of being mislabeled, a conclusion supported by a later sequencing run. These were retained in analyses of assignment strength, biological replication, and rarefaction but excluded from those relating to phenology and overall abundance of taxa, where their inclusion would have been misleading.

## Results

### Read Output after Filtering

**Fig 1:**
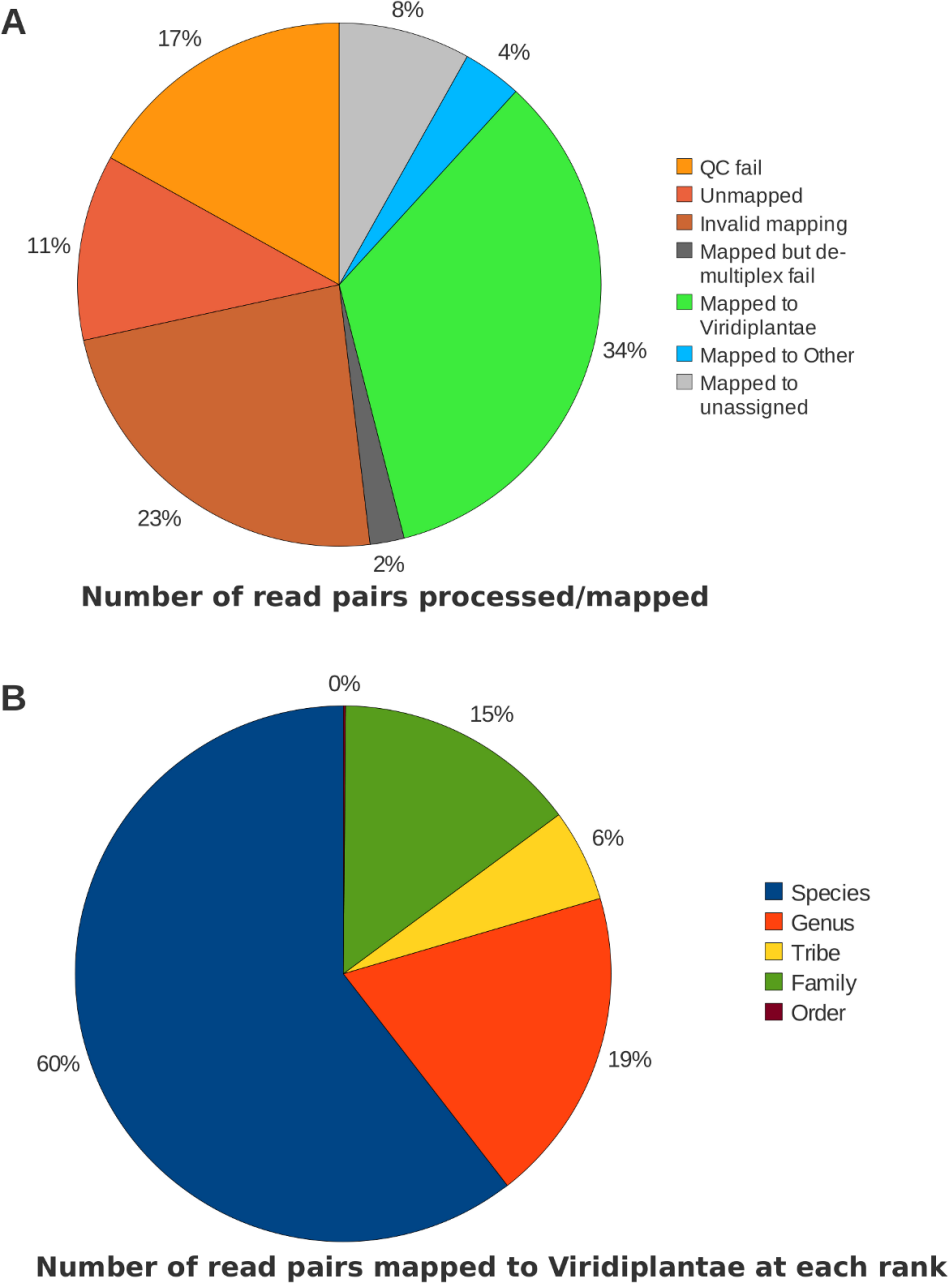
Pie charts summarizing sequencing output and read fate.

Initial sequencing output was 12.2 million read pairs (Fig 1A). Each read pair consisted of a “read 1” initiated by the ITS4 fusion primer and a “read 2” initiated by the ITS5a fusion primer; their mean lengths after trimming were 254.7 and 231.2, respectively. Unfortunately, we experienced a substantial drop-off in base quality in read 2 after approximately 150–200 cycles. This effect was observed across multiple pilot runs and in our hands frequently occurs in 600-cycle (2x300 bp) MiSeq applications regardless of template. This read degradation unfortunately led to the loss of over a quarter of read pairs when a 150-bp threshold length per read was applied. The mean number of mapped read pairs per sample was 58,372, with a coefficient of variation of 0.635. The failure rate for de-multiplexing was inferred to be 4.4% of mapped reads (~2% of total reads), based on the proportion of reads in the ‘undetermined bin’ mapping to OTUs of presumed eukaryotic origin and therefore not deriving from the phiX spike-in (a bacteriophage sequence added to final library preparation for calibration and improved platform performance).

### Number, Assignment, and Abundance of 97% OTUs

There were 7,696 97% OTUs, of which 4,610 could be given a taxonomic assignment under our criteria. A total of 173 distinct taxa were assigned, 128 of which were within the kingdom Viridiplantae (green plants) and the remainder mostly fungi. We limited our list of identified plant taxa to the 121 taxa with at least 50 total reads, an arbitrary minimum threshold chosen to suppress rare taxa arising from mapping error, which can occur at a low rate [53]. S2 File provides a spreadsheet of all taxonomic assignments and read counts by library. S3 File provides the corresponding FASTA file of OTU scaffolds. Many of the unassigned OTUs had strong but incompatible matches for each read of the pair (i.e., from distantly related taxa) suggesting that these were chimeras. This was substantially more than the 140 OTUs already removed as chimeras based on UCHIME results.

The OTU-averaged bit score and percent identities of BLAST HSPs are shown in Table 1 for all plant species assignments. Fig 1B shows the proportion of OTUs and mapped reads that were assigned at each of five taxonomic ranks within the Viridiplantae, as well as non-plant or unassigned OTUs. Roughly 60% of reads mapped to plants were assigned at the species level and another 19% of reads were mapped to the genus level, after binning all *Melilotus* OTUs as one species, *Melilotus officinalis* (see below), consistent with current taxonomic practice.

**Table 1:** Taxonomic assignments to species within Viridiplantae (green plants), sorted by the mean read counts per million (cpm).

Taxon	Mean bit score	Mean % identity	Min. cpm	Mean cpm	Max. cpm	CV	Species listed on state flora*	Genus listed on state flora*
*Melilotus officinalis*	798.6	98.3	8737.4	209000.6	767531.5	0.7	Y	Y
*Sonchus arvensis*	747.4	97.3	19.0	41274.6	255622.0	1.5	Y	Y
*Anemone canadensis*	788.5	97.9	0.0	34900.8	341125.1	2.2	Y	Y
*Artemisia absinthium*	904.3	98.5	0.0	23338.2	324582.2	2.7	Y	Y
*Cirsium arvense*	887.9	97.1	0.0	17932.3	241477.1	2.4	Y	Y
*Brassica nigra*	823.9	96.7	0.0	16021.8	319927.2	2.8	Y	Y
*Brassica oleracea*	891.3	94.8	0.0	13211.7	144256.8	2.3	N	Y
*Bassia scoparia*	909.6	98.6	0.0	11258.3	267961.5	4.0	Y	Y
*Helenium autumnale*	884.3	97.6	0.0	9226.3	221665.9	4.3	Y	Y
*Helianthus petiolaris*	838.5	93.2	0.0	7817.8	104791.5	2.7	Y	Y
*Raphanus sativus*	907.2	96.8	0.0	6023.3	209871.7	5.0	Y	Y
*Spartina pectinata*	912.7	98.8	0.0	5716.0	208647.5	4.2	Y	Y
*Zea mays*	915.1	98.1	0.0	5138.7	108385.1	3.7	Y	Y
*Brassica napus*	898	96	0.0	3520.0	39793.1	2.5	N	Y
*Elaeagnus moorcroftii*	835.2	98.1	0.0	2720.8	43779.5	3.2	Y	Y (as *E*. *angustifolia*)**
*Phalaris arundinacea*	829.6	97.3	0.0	2539.3	56050.6	3.2	Y	Y
*Cirsium vulgare*	897.5	97.2	0.0	2395.0	89652.4	5.4	Y	Y
*Cicuta maculata*	824.3	99.1	0.0	1865.5	166852.2	9.2	Y	Y
*Persicaria viscosa*	885.7	97.5	0.0	1837.8	52360.7	3.5	N	Y
*Ratibida peduncularis*	781.5	98.2	0.0	1811.4	98762.4	6.6	N	Y
*Sium suave*	905.3	98.5	0.0	1783.9	76437.8	5.2	Y	Y
*Brassica juncea*	823.9	96.4	0.0	1332.2	14822.7	2.0	Y	Y
*Bolboschoenus caldwellii*	856.5	93.6	0.0	1217.2	37829.4	4.4	N	Y
*Lactuca serriola*	754.3	92.4	0.0	1167.9	34928.8	4.7	Y	Y
*Dalea purpurea*	874.1	99.2	0.0	876.8	48457.0	6.3	Y	Y
*Convolvulus arvensis*	883.2	99.5	0.0	710.5	40295.2	6.0	Y	Y
*Monarda fistulosa*	888.8	98.8	0.0	706.8	45577.8	7.3	Y	Y
*Euphorbia esula*	866.7	97.1	0.0	482.3	9708.7	3.6	Y	Y
*Brassica carinata*	805.3	97.4	0.0	393.0	4728.3	2.1	N	Y
*Heterotheca villosa*	900.2	98.6	0.0	387.6	15687.5	5.2	Y	Y
*Actites megalocarpa*	696	95	0.0	310.4	2415.9	1.7	N	N
*Chamerion angustifolium*	838.9	97.3	0.0	268.4	25164.5	9.7	Y	Y
*Ceratodon purpureus*	925	95.9	0.0	263.7	5543.6	3.1	(bryophyte)	(bryophyte)
*Populus deltoides*	914.5	98.6	0.0	211.9	19342.6	9.4	Y	Y
*Helianthus praecox*	838	95.1	0.0	188.4	3145.8	2.8	N	Y
*Amorpha fruticosa*	702.7	98.1	0.0	152.2	6972.9	5.5	Y	Y
*Arctium lappa*	899	97.5	0.0	144.1	4536.4	4.4	Y	Y
*Imbribryum blandum*	883	92.5	0.0	109.6	9459.4	8.9	(bryophyte)	(bryophyte)
*Lonicera pilosa*	705	94.6	0.0	102.8	1389.4	2.2	N	Y
*Viburnum prunifolium*	862	96	0.0	87.1	8026.9	9.5	N	Y
*Helianthus pauciflorus*	797.5	98.1	0.0	74.0	1258.3	2.6	Y	Y
*Symphoricarpos occidentalis*	752	97.7	0.0	65.4	850.9	2.5	Y	Y
*Trifolium retusum*	859	98	0.0	65.0	3825.6	6.5	N	Y
*Lonicera japonica*	602	93.3	0.0	52.6	4526.5	8.9	N	Y
*Symphyotrichum novae-angliae*	806.3	96.1	0.0	49.3	3048.7	6.7	Y	Y
*Funaria hygrometrica*	913.5	94.1	0.0	42.0	3927.2	9.6	(bryophyte)	(bryophyte)
*Trifolium repens*	828	98.3	0.0	40.4	3149.0	8.1	Y	Y
*Lactuca sibirica*	724	95.8	0.0	36.4	1539.4	5.5	N	Y
*Helianthus occidentalis*	647	96.2	0.0	33.3	427.3	2.6	N	Y
*Eurybia divaricata*	790	93.4	0.0	25.1	727.8	5.0	N	Y (as *Aster*)***
*Pseudoroegneria tauri*	925	98.9	0.0	23.6	830.9	5.3	N	Y
*Symphyotrichum cordifolium*	881	97.1	0.0	19.6	858.1	5.6	N	Y
*Sinapis arvensis*	709	97.9	0.0	18.8	222.0	2.4	Y	Y
*Allium stellatum*	862.5	96.3	0.0	16.8	1253.0	7.7	Y	Y
*Phleum pratense*	925	96.9	0.0	14.1	1107.8	8.1	Y	Y
*Pleurozium schreberi*	910	98.7	0.0	12.2	459.2	5.3	(bryophyte)	(bryophyte)
*Elymus repens*	853	98.9	0.0	8.1	509.6	6.8	Y	Y
*Solidago houghtonii*	772	97.5	0.0	7.5	143.8	3.1	N	Y
*Laportea canadensis*	635	95.3	0.0	7.1	669.0	9.7	Y	Y
*Panicum queenslandicum*	762	92.5	0.0	6.9	349.8	6.6	N	Y

cpm = counts per million, CV = coefficient of variation.

*see text for sources.

**Elaeagnus moorcrofti is synonymous with *E*. *angustifolia*.

***Aster is currently considered strictly Eurasian. North American relatives have been assigned to new genera including *Eurybia* [80].

### Plant Taxa Identified

Species-rich families containing known forage plants [58,59] were prominent, such as the Asteraceae, Brassicaceae, and Fabaceae (Table 1, S2 File). We detected a small abundance but unexpected diversity of non-crop graminoids. Some of these species (e.g. *Bulboschoenus caldwelli*, *Phalaris arundinacea*, *Spartina pectinata*) are facultative or obligate wetland plants, and together with other wetland associated monocots (*Sagittaria*, *Alisma*) suggest that the wetlands characteristic of the region were visited by pollen foragers. Other graminoid genera identified include typical prairie grasses of the northern Great Plains, such as *Elymus*, *Pseudoregenaria*, and *Panicum* [60]; counts per million (cpm) across all 96 samples were highly correlated for each of the three pairwise comparisons of these species (Spearman’s R^2^ > 0.7, P << 0.001), suggesting a co-flowering community. None of these graminoid species had cpm that were correlated with *Zea mays*, an abundant graminoid crop known to be visited by honey bees [61], indicating that mis-assignment of *Z*. *mays* pollen is not a plausible explanation of these apparent pollen sources. However, contamination of foraged pollen by wind-borne pollen is a possible alternative to active collection.

Erroneous taxonomic assignments may occur for several reasons, including imperfect taxonomic assignment of GenBank sequences and an inability to accommodate all ambiguity in BLAST matches with a single set of LCA parameters. Post-hoc inspection and assessment of OTU assignments is therefore recommendable to confirm unexpected assignments and to identify causes of unexpectedly deep taxonomic assignments. In four cases, post-hoc analysis led us to filter BLAST output or alter assignments. In the first case, preliminary analysis in Megan indicated that the majority of assignments at the “Eukaryote” node were due to the default inclusion of “environmental sample” in the taxonomy. Inspection of BLAST reports showed that OTUs with “environmental sample” as a match frequently had matches of comparable strength to asterid genera such as *Symphyotrichum* and *Eurybia*. We therefore removed all BLAST matches (technically, all accessions that formed HSPs with an OTU) that contained “environmental sample” in the associated taxonomy, because these reference accessions do not represent a known taxon and obscure other plausible sources. The second case was the assignment of eight OTUs to the tundra grass *Dupontia fisheri*. These OTUs were re-examined and found to match the common temperate grass genus *Alopecurus* at a generally higher percentage identity, but HSPs had lower bit scores because the matched *Alopecurus* accessions were shorter. The mean percentage identity was 96.0% for *Alopecurus* HSPs (the highest mean of all genera matched by these OTUs) vs. 94.9% for *Dupontia* HSPs, whereas the mean bit scores of HSPs were 397.6 and 407.2, respectively. We therefore re-assigned all *D*. *fisheri* OTUs to *Alopecurus*, although the combined counts for this genus remained below our 50-count threshold for this data set. In the third case, the Asian species *Dioscorea polystacha* was initially identified as an OTU assignment. Re-examination of these OTUs revealed that only one *Dioscorea polystacha* accession was an HSP and all other HSPs were with accessions of a phylogenetically very distant species, *Raphanus sativa*, which is widespread in North America. Submission of this accession (FJ860088.1) to BLAST recovered no other hits to *Dioscorea* or any other monocot, only to the dicot genus *Raphanus*. We therefore filtered all HSPs to accessions of *Dioscorea*. The taxonomy of this accession has since been modified by NCBI to “unverified”. Finally, OTUs initially assigned to the NCBI species-level taxon “Helianthus sp. DH-2012” [62], which based on BLAST results appears related to other North Dakota *Helianthus* such as *H*. *petiolaris*, were re-assigned to the genus *Helianthus*. This was because it is unclear whether *Helianthus* sp. DH-2012 should be considered a distinct species or subsumed under another described *Helianthus* species.

**Fig 2:**
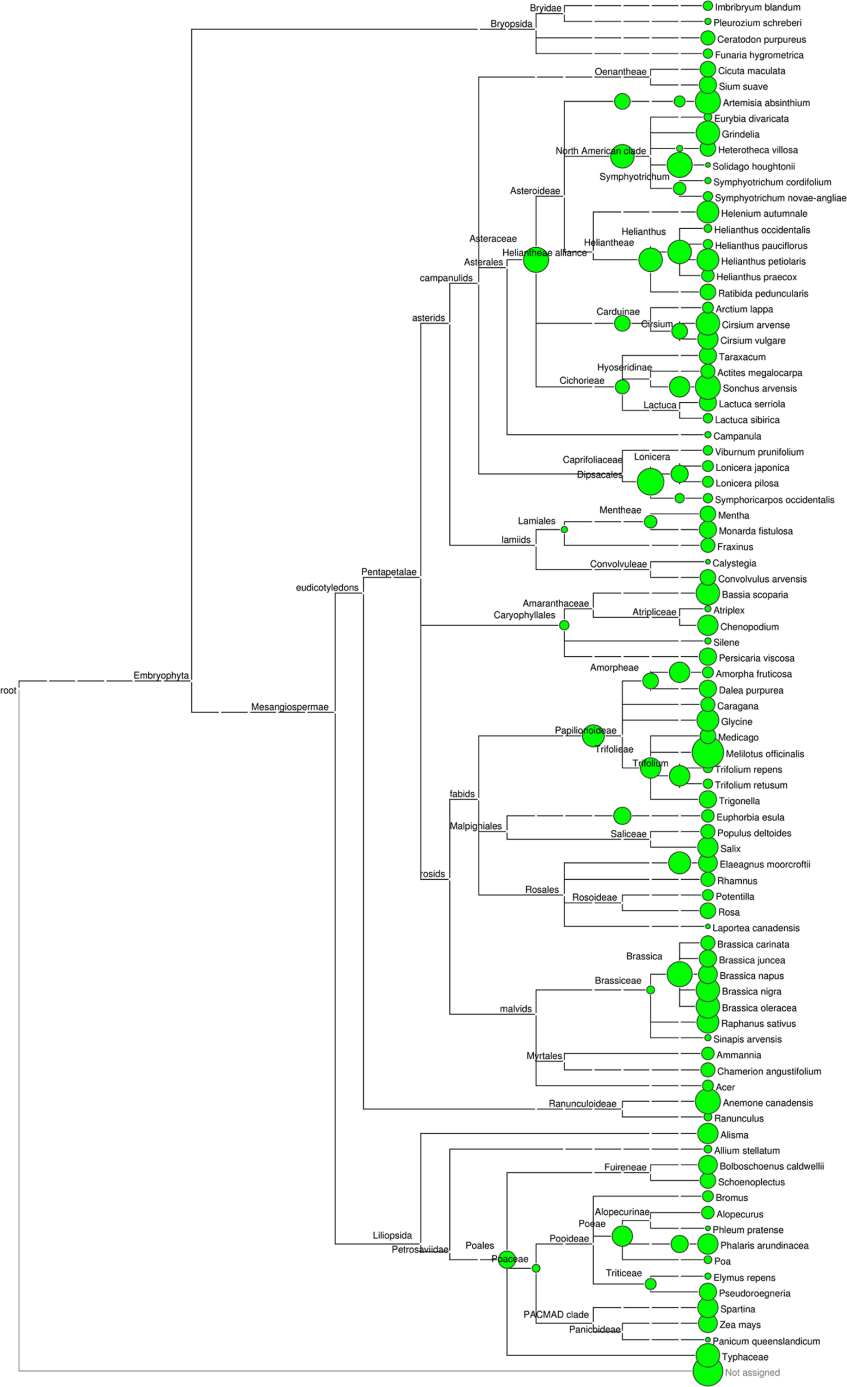
Relative read counts summed across all OTUs for each plant taxonomic assignment. The counts are represented in phylogenetic context with the size of the circle at each node proportional to the (square-root transformed) counts per million (cpm) reads across all samples combined. Assignments were made at five taxonomic ranks as described in the methods. Counts for interior nodes reflect progressively greater taxonomic uncertainty in the underlying OTUs; they do not represent sums of counts attributed to the leaves of that node.

Fig 2 shows the log-transformed read counts (cpm across all samples combined) of all plant taxa identified in phylogenetic context. *Melilotus* (sweet-clover) was by far the most abundant genus when summed across all samples. As previously noted, *M*. *officinalis* and *M*. *alba* are very closely related and considered a single species in some treatments, e.g. [46]. In general, low genetic divergence and/or incomplete lineage sorting of the (multi-copy) ribosomal locus are factors that potentially limit species-level assignment, regardless of the absolute score of matching reads [42]. GenBank accessions for these two species as well as *M*. *dentatus* showed a percent pairwise divergence of less than 1% (S4 File) and a maximum likelihood phylogeny indicated no support for monophyly (S5 File). It is therefore impossible to differentiate these species under any LCA parameters for this locus. We therefore consider all counts assigned to these species as *M*. *officinalis*.

Other genera with high total read counts included *Brassica*, *Anemone*, and a number of asterids including *Sonchus*, *Artemesia*, *Solidago*, and *Cirsium*. A few bryophytes were identified at moderate to low levels. As we are unaware of any active collection of spores from bryophyte sporangia, we hypothesize that spores adhere incidentally to body hairs during water foraging.

Considering only OTUs from plant taxa with species-level assignments and total read counts above the 50-count threshold, the average number of plant species with read counts > 0 in a sample was 23.0 (+/- 5.5). The diversity of taxa recovered per sample, quantified using the generalized approach of [56] of order 1, averaged 3.3 +/- 2.2.

### Concordance of Assignments with Geography

To assess the consistency of OTU assignments with plant distributional data, we used three extensive species lists for North Dakota: the USDA PLANTS database [46], the North Dakota Flora electronic checklist [44], and the phenology records compiled by [45] for the Fargo vicinity. All flowering-plant assignments (i.e., excluding bryophytes) were compared to these sources at the species and genus level. The proportion of all species-level assignments that were reported to occur in North Dakota was 64% (36 of 56, Table 1). Some of species assignments not present on these lists are nonetheless commonly cultivated plants such as *Brassica oleracea*, *B*. *napus*, and *Z*. *mays*. The proportion of genus-level assignments that were reported to occur in North Dakota was 98% (55 of 56). The single exception was *Actites megalocarpa*, endemic to Australia but related to North American genera. Only one OTU was assigned this taxonomy, and the total number of reads mapping was 97. In all other instances where *A*. *megalocarpa* was an HSP of an OTU, the actual taxonomic assignment made was *Sonchus arvensis* or *Sonchus*, of the same subtribe (Hyoseridinae) and a known honey-bee pollen source [63]. The Pearson R^2^ between *A*. *megalocarpa* cpm and *S*. *arvensis* cpm was 0.90. While the erroneous assignment and likely true source of the reads are easily diagnosable in this instance, whether the underlying cause was methodological or a phylogenetically discordant ITS fragment in *Sonchus* populations is unclear. This incorrect assignment illustrates the need for reasonable count thresholds and for conservative taxonomic assignments when independent sources of validation are minimal.

### Read-Count Concordance with Phenology

**Fig 3:**
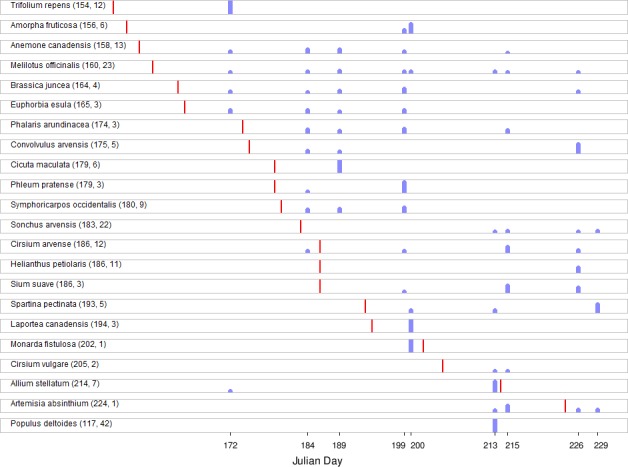
The proportion of total counts per million mapped reads (cpm) by sampling date for plant taxa with phenological records in [45] that overlap the range of pollen sampling dates. Red bar shows mean Julian date of first flowering from [45] relative to sampling dates (blue bars). Values in parentheses following the species name are the mean date of first flowering and the number of observations underlying that mean, respectively. Three sampling dates after Julian day 228 are omitted to avoid compressing the horizontal axis unnecessarily, as no first flowering dates occurred in that time frame for species in our sample. No red bar is shown for *Populus deltoides* because that mean day of first flowering is outside the range of the horizontal axis; this was also done to avoid compressing the axis.

We used an extensive record of first flowering times of North Dakota plants [45] to assess the concordance of pollen assignments with expected flowering time. These data were compiled as rows of species with columns containing first observed flowering date for a series of years dating back to 1910, although the number of years of observation varies among species (range 1–42 years for species assignments in this study). We calculated the proportion of counts for each of 22 taxa identified in our samples for which flowering data were available and plotted these versus the mean Julian day of first flowering (Fig 3). While the concordance is not exact, substantial proportions of total counts for that species (i.e., > 5%) were generally not detected much prior to expected first flowering (in some cases, much later, e.g. *Helianthus petiolarus*). However, *Populus deltoide*s was identified in sequence reads from summer samples (~Julian day 210), well after the expected first bloom (see Discussion).

### Biological Replication and Rarefaction

**Fig 4:**
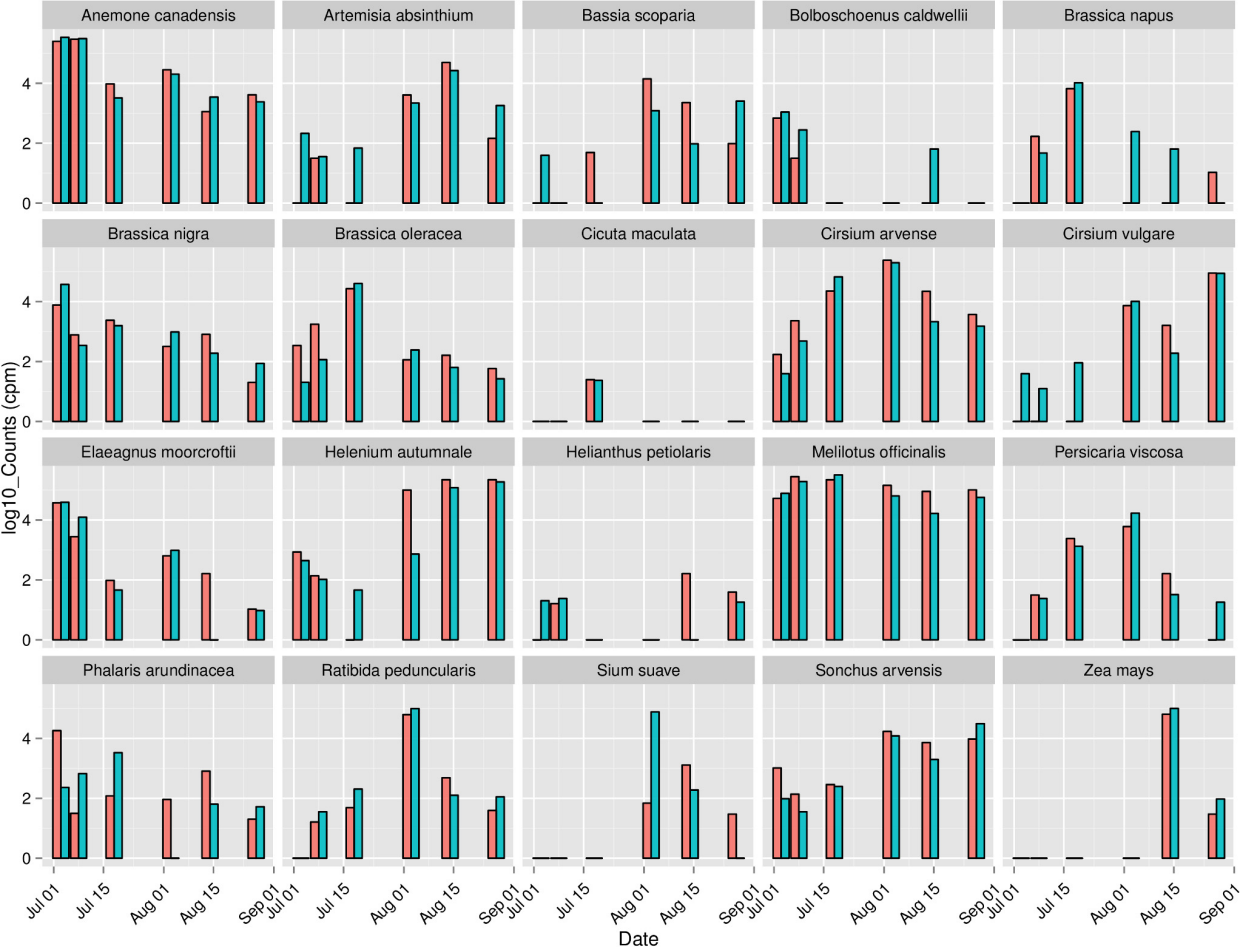
Forage calendar for two honey bee colonies (red and blue bars) located in Apiary 3 in 2009, highlighting the temporal progression of plant taxa. Combined OTU counts for the 20 most common plant species-level assignments, based on the summed counts for all apiaries in 2009, are represented. Vertical axis represents the log_10_ transformed counts per million mapped reads (cpm) for each taxon at each sampling point.

**Fig 5:**
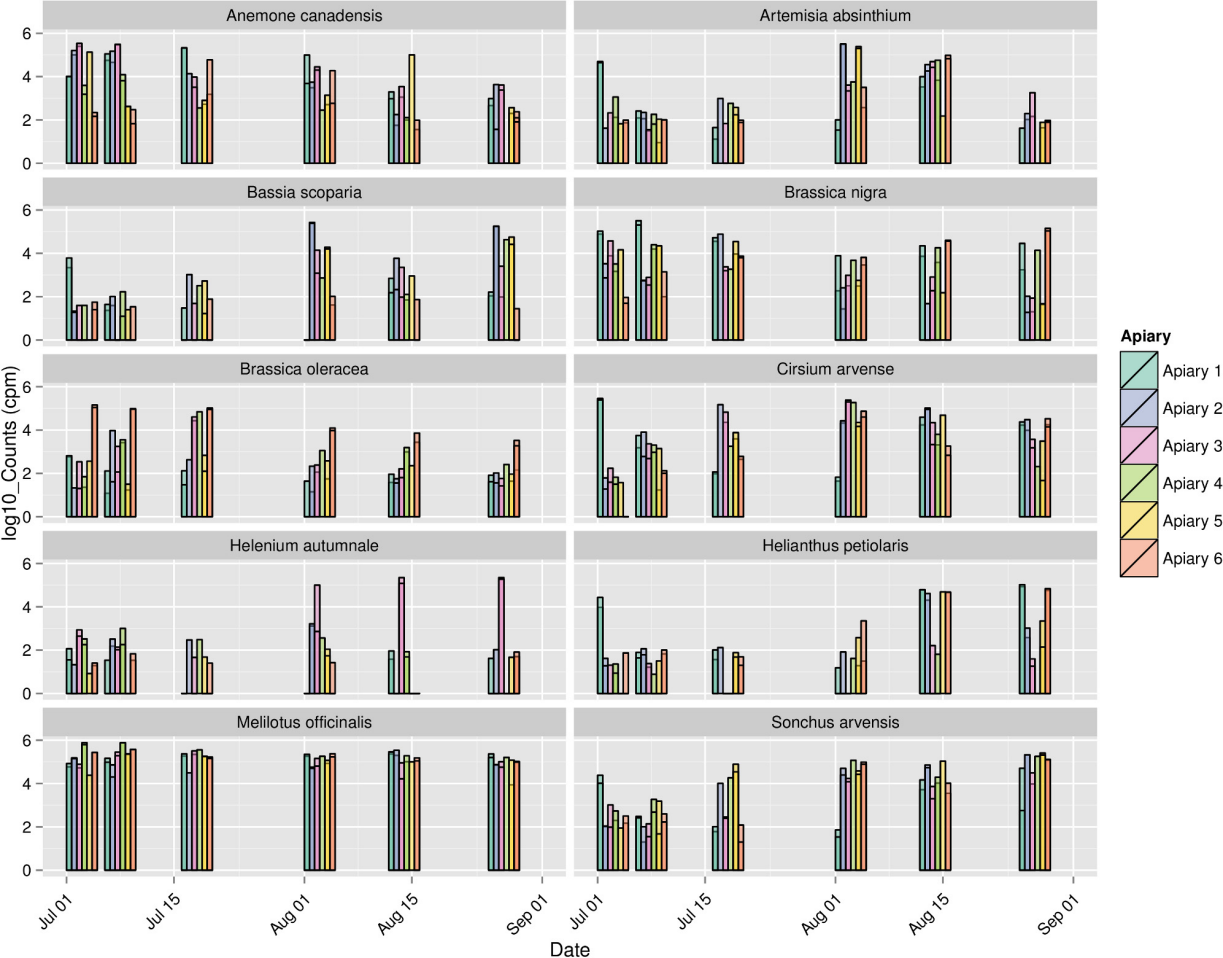
Forage calendar highlighting variation in pollen collection among 6 apiaries in 2009. Combined OTU counts for each of the 10 most common plant species-level assignments, based on the summed counts for all apiaries in 2009, are represented. Vertical axis represents the log_10_ transformed counts per million mapped reads (cpm) for each taxon at each sampling point. Vertical bars are transparent to show data for multiple colonies sampled within the same apiary.

**Fig 6:**
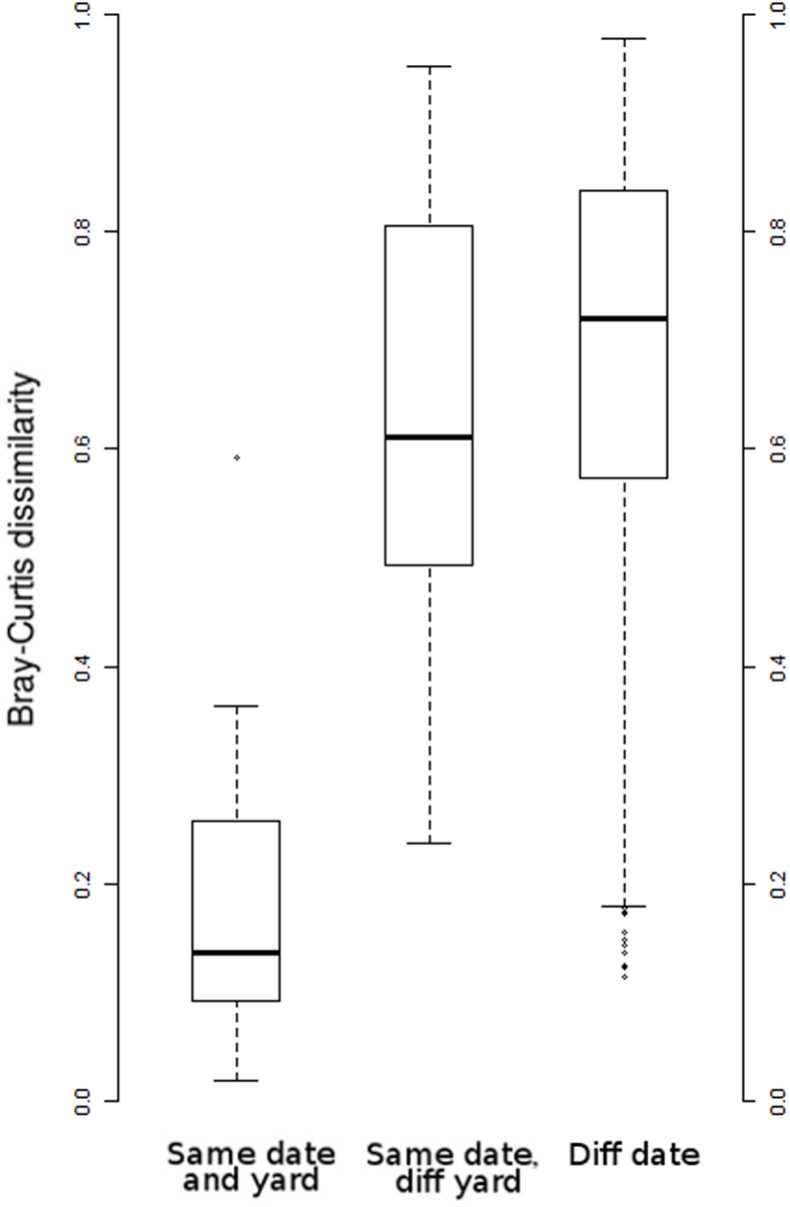
Box plots of pairwise Bray-Curtis dissimilarities computed with Megan v. 5.10.2 [52]. The range of possible values is 0–1. Only 2009 samples were compared as relatively few 2010 biological replicates were available and a year-effect on phenology is possible. Samples collected from different hives but from the same date and apiary were more similar than samples collected from different dates and apiaries. This indicates that variation among biological replicates is lower than spatial or temporal variation in foraging.

Fig 4 shows log-transformed counts of the top 20 plant species-level assignments for Apiary 3, while Fig 5 shows counts of the top 10 assignments for all apiaries in 2009. These subsets of the data were chosen to represent the overall consistency among sites in the phenological progression of taxa while avoiding overly dense figures. Fig 5 also demonstrates clear differences in the relative abundance of taxa among sites. That is, we observe a high degree of similarity between biological replicates within an apiary and a gradual shift of taxon abundance between successive samplings in the same apiary, whereas greater differences occur among apiaries in different landscapes. S6 and S7 Files provide the equivalent comparisons for the top 20 plant taxa regardless of taxonomic rank assignment. The similarity of biological replicates can be compared quantitatively with the Bray-Curtis distance, an appropriate measure for read counts [55]. Colonies samples from the same apiary and date in 2009 (i.e. biological replicates) were much more similar to each other than samples from different apiaries or dates (Fig 6). We included only 2009 samples in this comparison because few replicates were available for 2010 and because comparisons across years may not be valid due to phenological variation or changes in plant communities. Note that while hives of the same apiary are sited near each other in a landscape, foraging behavior develops through colony-level decision making and is influenced by hive parameters such as brood production and stores. Thus, it is not expected that neighboring hives should exhibit identical forage behavior. Nonetheless, our results support the view that landscape factors strongly dictate honey bee foraging patterns.

**Fig 7:**
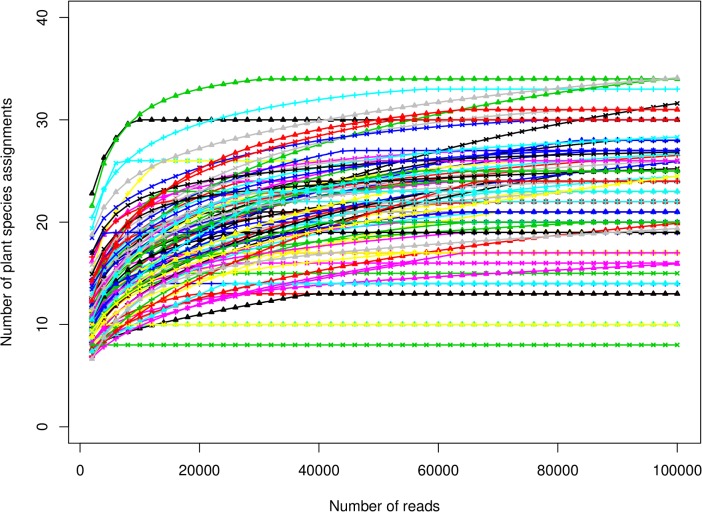
Rarefaction curves for all samples. Only species-level assignments within Viridiplantae are considered, with all other assigned or unassigned counts combined into a single ‘other’ bin. Subsamples ranged from 2,000 to 100,000 reads per sample, subject to the limit of actual sample size.

Rarefaction can be used to assess whether sequencing depth was adequate at this large scale of multiplexing to recover the diversity within samples. However, given counts assigned to hierarchically structured taxa, it is necessary to select a taxonomic rank as the unit of classification. Fig 7 shows rarefaction plots of all 96 samples for counts assignable to species within Viridiplantae, with non-plant species, higher-level assignments, and unassigned reads aggregated into a single ‘other’ bin. The figure shows that for most samples, a relatively complete list of species-level assignments is recovered with roughly 50,000 reads; this read depth was achieved for 55 of 96 samples (57%).

### Assignment Rank, Score, and Percent Identities Indicate Database Gaps

While OTUs were assigned to taxa based on a common LCA criterion, the actual alignment similarities between OTUs and GenBank accessions nonetheless vary by taxon. We tabulated the mean percent identities of HSPs between each OTU and matching accessions of the taxon to which that OTU was assigned (averaged over read pairs and then over OTUs). Plotting these values phylogenetically provides some additional insight into factors affecting taxonomic assignment (S8 File). Tips with lower percent identity are likely to represent OTUs for which the true origin is not well represented in the reference database, for example, OTUs assigned to *Calystegia* and to *Typhaceae*. These assignments are relatively distant from any other nodes with strong assignments, and thus are presumably incorrect even though they meet the assignment criteria. Analysis of these examples in light of local flora and inferred phenology should help guide voucher sequencing that may ultimately recover a stronger match for these OTUs.

### Non-Plant Taxa Identified

Environmental microbes including plant-associated fungi, contributed a modest proportion of OTUs and mapped reads (S2 and S9 Files), as has been observed in other studies [37]. An exception was *Ascosphaera apis*, a pathogenic fungus causing “chalkbrood disease”, which was unexpectedly the second most abundant taxon overall in the data set. Most of the *A*. *apis* reads come from a restricted set of colonies and time points (S10 File) and are likely from spores contaminating the pollen after it arrives at the hive, since successful *A*. *apis* infections produce huge numbers of spores that can accumulate in under-hive pollen traps or be borne on the bees themselves [64]. This is corroborated by our occasional observation of chalkbrood “mummies” (dead larvae visibly infected with *A*. *apis*) in the pollen traps. Although we removed such obvious detritus from sampled pollen prior to storage, it is likely that *A*. *apis* spores remained in the sample and indeed, their complete removal would be infeasible. Future work will investigate the use of blocking primers that suppress the amplification of *A*. *apis* ITS sequence, so that localized outbreaks of the pathogen do not absorb a large number of reads.

Other known honey-bee associates that were detected include *Bettsia alvei*, a fungus associated with spoiled pollen in hives [65]. A low level of reads mapped to a devastating parasite of honey bee, the mite *Varroa destructor* (*Varroa jacobsoni* in the NCBI taxonomy, see [66]), which presumably derived from tissue of dead mites accumulating in the under-hive traps. More surprising was the fact that we did not detect contamination from honey bees themselves.

## Discussion

Developing novel methods for understanding pollinator foraging and plant-pollinator interactions is timely considering heightened societal concern over declining pollinator populations and the general consensus among the scientific community that forage limitations play a role in these declines [6]. Recently, there has been a spate of publications demonstrating the utility of high-throughput barcode sequencing to generate species profiles of bee-collected pollen, illustrating a convergence of technology and need in multiple fields [20–23]. While these methods represent great gains in efficiency, it remains challenging to achieve species-level identifications, an important objective for assessing the quality of landscapes on which honey bees are housed and identifying seed mixes that enhance honey bee nutrition. Here we have achieved a high rate of species-level assignments by using non-overlapping read pairs of the nuclear ribosomal ITS region, omitting the uninformative 5.8S region. However, the LCA approach does not produce a probabilistic estimate of confidence in OTU assignments or read mappings, and it is clear from S7 File that some species-level assignments are dubious based on percent identity of HSPs and biogeography (e.g., the Australian species *Panicum queenslandicum* with a mean percent identity of <93%). Furthermore, the consequences of different bioinformatic processing steps and confidence in the resulting assignments remain difficult to quantify [67] (but see [20]). Keller and colleagues [20] used a training-set approach with uniform-coverage ITS2 sequences to assess assignment reliability, and estimated that 96% of genus-level assignments and 70% of species-level assignments in their study were correct based on simulations. Complete ITS references of the regional flora where our study was performed would likely allow comparable or greater performance given the greater locus coverage used here, yet we remain far from that ideal. For example, of 748 species listed in the phenology record of [45], only 433 were represented in the ITS sequences we initially downloaded from GenBank to seed our clustering strategy (see Methods), an estimated 57.8% rate of at least partial sequence representation.

While non-overlapping paired-end sequencing has allowed us to take fuller advantage of the ITS region for taxonomic assignment, there are potential bioinformatics costs of this strategy including higher sequencing error compared with double-interrogated bases and more complicated workflows. However, considering the relatively low error rate of the Illumina platform under ideal conditions (~0.1% [68]) and the importance of match length (bit score) in taxonomic assignment, we believe maximizing the total number of bases is of much greater importance. Read pairs do not need to overlap to have a fully described alignment relative to a reference sequence, including orientation and insert size constraints, and we see no reason why chimera detection should suffer decreased sensitivity. Indeed, paired-read sequencing of long clones performed well in a simulation study [69]. Even so, chimeras inferred from discordance between paired-read BLAST matches far exceeded those filtered with uchime, and represent a substantial loss of efficiency, a problem intrinsic to mixed-template PCR [70].

Not surprisingly, given the varied nature of the uncurated GenBank database, no single set of LCA criteria we investigated was free of dubious assignments when investigated post-hoc. Sources of database error may include submitter error, outdated or ambiguous terms in the taxonomic scheme, and unequal sequence representation of taxa (see above). Continued growth of curated barcode databases will help alleviate these problems, but the multicopy ITS region in particular suffers from the potential for incomplete lineage sorting and thus conflation of closely related species regardless of LCA criteria [42]. While it is often presumed that all ITS copies are homogenized within a taxon by gene conversion [71], the generality and pace of this occurrence is not well documented [72], and could be complicated by factors such as hybridization, polyploidy, and pseudogenization [42]. The ITS regions are therefore suboptimal for discriminating closely related species, but appear efficient for genus-level assignments and are well-established in public databases. While we chose ITS in part because chloroplast retention in pollen varies by species [38,39], potentially leading to taxonomic dropout, the results of [22] with trnL effectively dispel such concerns.

Inferred pollen foraging by honey bees in our study closely mirrored plant distributions and phenology for Eastern North Dakota [73]. In June, bees collected pollen from several different genera of woody plants and early season forbs. In July and August, honey bees collected pollen from multiple genera of forbs and graminoids. *M*. *officinalis* was detected in high abundance throughout the growing season in all apiaries and it is well known that North Dakota beekeepers target field patches of *Melilotus* for honey production. Generally, the few discrepancies had plausible biological interpretations. For example, an apparent discordance between flowering time reported by [45] and our results occurred for *Artemisia absinthium*. Our analysis revealed honey bees collected *A*. *absinthium* pollen in early- to mid-July, whereas the single observation of first flowering time for this species in [45] was August 12, 1910. However, other sources for North Dakota (e.g., [74]) indicate that *A*. *absinthium* typically begins flowering in late July, which is more consistent with our data. The large lag between first flowering and our first detection of *Amorpha fruticosa* probably indicates an erroneous assignment of pollen actually deriving from its congener, *A*. *canescens*, which also occurs in the region, is visited by honey bees [59,75], and had a mean Julian flowering date of 187 versus 156 for *A*. *fruticosa*. The two species share approximately 99% nucleotide identity at ITS sequences based on BLAST results. A similar situation may have occurred with *Allium stellatum*, in that two widely spaced foraging periods are evident, yet the closely related congener *A*. *cernuum* is reported to occur in cooler, moister habitats [76] and other North Dakota *Allium* species are not closely related by BLAST analysis. Another noteworthy discrepancy is the tree *P*. *deltoides*, observed in sequence reads from summer samples (~Julian day 210) long after the expected first bloom. Interestingly, *P*. *deltoides* is the preferred species for foraging of propolis, tree resin collected by bees to seal hive gaps and which potentially improves colony resistance to disease [77]. We hypothesize that resin from *P*. *deltoides*, which is also carried on the corbicula, contained the source DNA that was carried over to pollen. The timing of the detection of *P*. *deltoides* may have coincided with the addition by apiary managers of “supers”, specialized boxes added to a hive to stimulate foraging and honey storage. Addition of supers can concurrently stimulate propolis collection in order to seal gaps between the super and established hive boxes.

Overall, our results indicate sufficient precision and taxonomic recovery to relate foraging patterns to individual honey bee colonies throughout the growing season. This was achieved without recourse to a tailored reference database of plants identified *a priori*, but rather with *de novo* OTUs that were taxonomically characterized by comparison to a highly inclusive and loosely curated database of ITS sequences. Even microbial species that were incidentally recovered did not appear as random draws of environmental taxa, but rather showed a predominance of bee commensals and known plant pathogens. The relative success of the method under these conditions, in terms of consistency and concordance with previous apidological research, bodes well for its application to other species or environments where floral resources are less well documented or more diverse. Even so, we consider it a natural refinement of the approach to gather additional voucher sequences based on initial results, and to use distributional, phenological, or other constraints to better represent the local ITS sequence space. Indeed, we were surprised by the detection of wetland-associated plants in this work, particularly graminoids that are presumed to be predominantly wind-dispersed, and propose additional voucher sequencing and corroborative observations to understand the scope and conditions under which these plants are visited by honey bees.

While quantitative comparisons of read counts are likely to be consistent given adequate coverage, as assessed by Bray-Curtis dissimilarities of biological replicates and by rarefaction analysis, and the values are reasonable based on published and communicated honey bee observations [59] we cannot currently demonstrate the degree to which read counts from a complex sample correspond to the proportion each taxon was actually present in each sample. There are potential biases related to ITS copy number (see for example [78]) per unit mass of pollen, extraction efficiency, and amplification efficiency [70]. One way to adjust for these differences is to assess the efficiency of ITS capture for a pure species sample relative to control mixtures. This could feasibly be done for tens of species of interest, but interaction effects would most likely have to be disregarded. Nonetheless, as long as biases remain consistent, the relative composition of samples should be tractable quantitatively (e.g., differences in sample similarity or diversity metrics associated with variables of interest), but because PCR is an exponential process, it may be prudent to moderate the variability of count data with square-root or log transformation for modeling purposes. Control mixtures, which were not available for this study because of the sampling method implemented, will be investigated in future work, as will comparisons with other palynological methods such as microscopy. To date, comparisons of micropalynology and metagenetics indicate that the latter is effective in identifying pollen sources, but read counts may not be consistently related to visual counts. For example, [22] were able to identify six grass species in a control mixture with metagenetic sequencing of the trnL chloroplast locus, whereas these species were not distinguishable by microscopy. They also analyzed airborne pollen collected in traps with both approaches and found a general quantitative agreement between the two, although greater taxonomic diversity was detected genetically. Kellerand colleagues [20] found numerous species were only detectable by one of two approaches, ITS2 sequencing or micropalynology, but when both methods identified a species the (log-scaled) quantities were well correlated. On the other hand, [23] found a generally poor correlation between species proportions using similar methods.

Additional work is needed to distinguish pollinator preference from resource availability. However, some initial indications of preference are suggested by phenology plots in Fig 3. For example, some plant species show little lag between inferred first flowering date and maximum collection (e.g., *Monarda fistulosa* and *Laportea canadensis*), whereas other species showed a substantial lag (e.g., *Convovulus arvensis* and *S*. *arvensis*). Thus, with sufficient observational data of plant flowering, it should be possible to assess the extent of divergence of realized foraging from background availability, and how this may depend on intra- and interspecific competition.

In addition to technical efforts to achieve greater read length and quality and to suppress microbial ITS sequences, a variety of future research directions are apparent. Honey bees forage for pollen throughout the growing season, over a 50–100 km^2^ area [35]. Thus, pollen collected from honey bee colonies represents a broad sampling of floral resources available across a landscape. Identification of bee collected pollen could be used to detect particular plants of interest in a landscape, including invasive or regulated plants. Furthermore, pathogen loads are potentially quantifiable, with respect to both plant and insect diseases. For example, our results demonstrate that a pathogen such as *A*. *apis* can be readily detected and tracked through time. Genetic analysis of bee-collected pollen will allow researchers to quantify how land-use conditions surrounding apiaries affect plant species diversity and floral resource quality. Forage information gleaned from pollen identification from multiple apiaries could be used to inform habitat restoration and conservation efforts. Application of this technique may be particularly important in North American temperate grasslands that have undergone significant land-use change in recent years [79]. The U.S. government has recently initiated a national strategy for enhancing pollinator health and will restore or enhance 7 million acres of land as pollinator habitat [10]. Results from honey bee foraging studies could be used to inform seeding mixes for grassland restoration efforts. Annual sampling of bee-collected pollen could be useful for understanding how climate change affects plant phenology and distributions. If pollen were collected from bee colonies at sufficient intervals, then floral resource calendars could be developed to highlight the flowering period of different plants and how flowering potential changes over long temporal periods. Currently, phenological mapping of flowering times is often done by direct field observation or remote sensing [73]. Shifts in plant species’ ranges could also be documented via identification of bee-collected pollen from multiple years. Native pollinators, particularly native bees, are likely amenable to pollen profiling as DNA extraction and PCR amplification requires small amounts of input tissue.

## Supporting Information

S1 File Map and summary of hive locations and sampling events. Note that duplicate pollen samples were collected from 2 colonies from Apiary 1 on July 19, August 1, and August 17, 2010.
(PDF)

S2 File Spreadsheet of all taxonomic assignments and total read counts by sample. (XLS)

S3 File Fasta file of OTUs. Taxonomic assignment, if any, is stated in the sequence header.
(TXT)

S4 File Percent identity matrix of aligned accessions of three species of *Melilotus*. (XLSX)

S5 File Maximum likelihood phylogeny of the aligned *Melilotus* sequences from S4 File. (PDF)

S6 File Forage calendar for Apiary 3 in 2009, highlighting the temporal progression of plant taxa. Combined OTU counts for the 20 most common plant assignments are represented, regardless of taxonomic level assignment. Vertical axis represents the log_10_ transformed counts per million mapped reads (cpm) for each taxon at each sampling point.
(PDF)

S7 File Forage calendar highlighting variation among apiaries in 2009. Combined OTU counts for the 10 most common plant assignments are represented, regardless of taxonomic level assignment. Vertical axis represents the log_10_ transformed counts per million mapped reads (cpm) for each taxon at each sampling point. Vertical bars are transparent to show data for multiple colonies sampled within the same apiary.
(PDF)

S8 File Mean percent identity of HSPs associated with taxa at each assigned node of the flowering plant taxonomy, in phylogenetic context. The phylogram was created with Megan v. 5.10.2 [52] using dummy counts appropriate to scale each node according to the legend.
(TIF)

S9 File Phylogram created with Megan v. 5.10.2 [52] depicting counts per million mapped reads (cpm) for non-plant taxa, in phylogenetic context. The area of each pie is proportional to log_10_(cpm), and each pollen sample is represented by an arbitrary color in order to illustrate that most taxa other than *Ascosphaera apis* were detected in few samples.
(PDF)

S10 File Distribution of log10 counts per million mapped reads (cpm) for the fungus *Ascosphaera apis*, a honey-bee pathogen. A background level of *A*. *apis* was found in all apiaries as well as apparent outbreaks in individual apiaries.
(PDF)
