# Supplementary material for: Taxonomic Characterization of Honey Bee (Apis mellifera) Pollen Foraging Based on Non-Overlapping Paired-End Sequencing of Nuclear Ribosomal Loci
Source: PLoS One. 2015 Dec 23;10(12):e0145365. doi: 10.1371/journal.pone.0145365 (PMC4689544; doi:10.1371/journal.pone.0145365)
Supplement: S1 File — Note that duplicate pollen samples were collected from 2 colonies from Apiary 1 on July 19, August 1, and August 17, 2010. (PDF) [file pone.0145365.s001.pdf]

Supporting Information File 1. Apiary locations, sample dates, and replicate number.

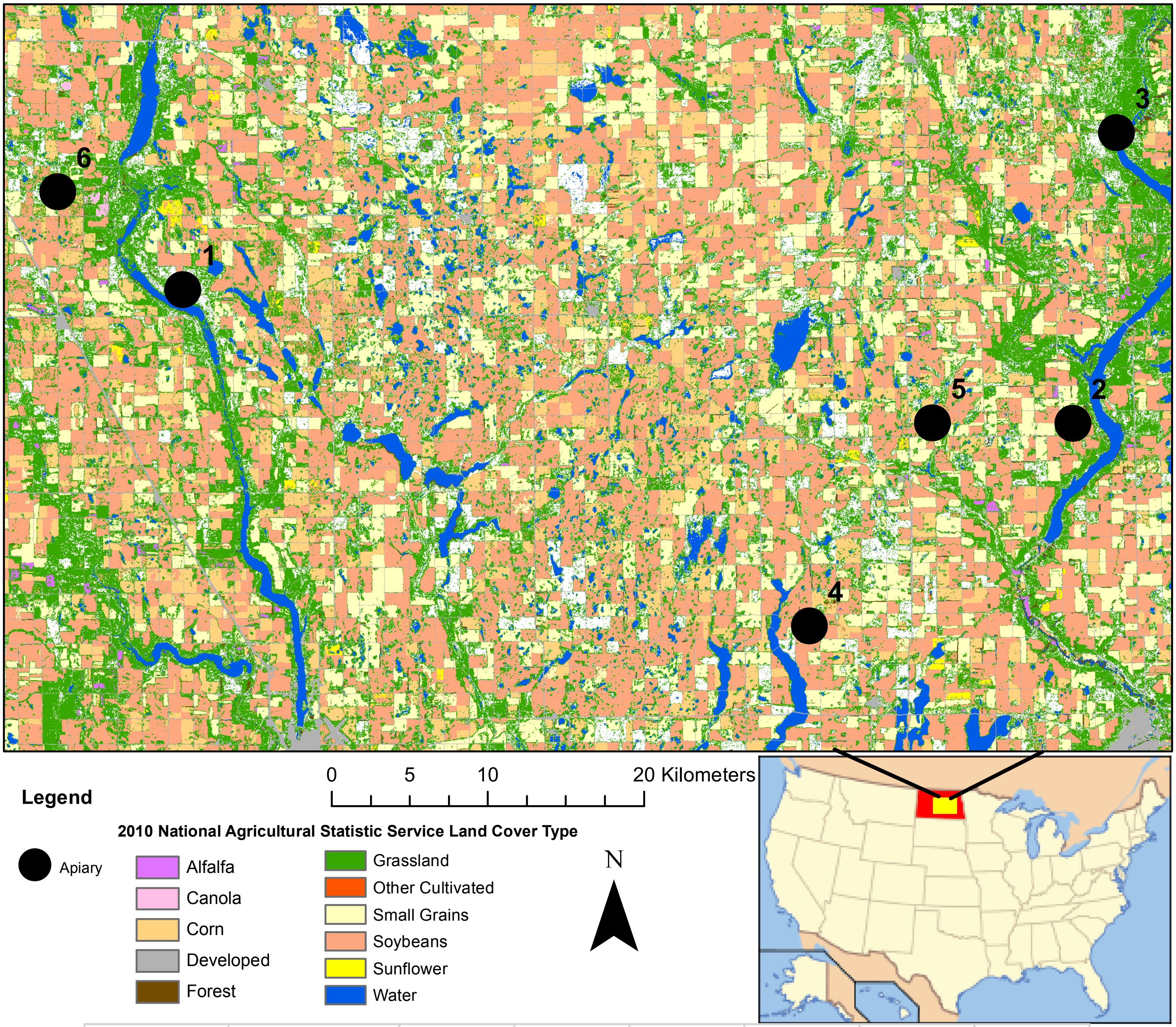

Julian Day	Calendar Day	Apiary 1	Apiary 2	Apiary 3	Apiary 4	Apiary 5	Apiary 6
184	Jul 3, 2009	2	2	2	2	1	2
189	Jul 8, 2009	2	2	2	2	2	2
199	Jul 18, 2009	2	1	2	1	2	2
215	Aug 3, 2009	2	1	2	2	2	2
226	Aug 14, 2009	2	2	2	2	1	2
239	Aug 27, 2009	2	1	2	2	2	3
172	Jun 21, 2010	3	0	3	0	0	0
200	Jul 19, 2010	4	0	3	0	0	0
213	Aug 1, 2010	4	0	3	0	0	0
229	Aug 17, 2010	4	0	1	0	0	0
237	Aug 25, 2010	2	0	0	0	0	0
251	Sep 8, 2010	2	0	0	0	0	0
