# Supplementary figures and images for: Taxonomic Characterization of Honey Bee (Apis mellifera) Pollen Foraging Based on Non-Overlapping Paired-End Sequencing of Nuclear Ribosomal Loci
Source: PLoS One. 2015 Dec 23;10(12):e0145365. doi: 10.1371/journal.pone.0145365 (PMC4689544; doi:10.1371/journal.pone.0145365)

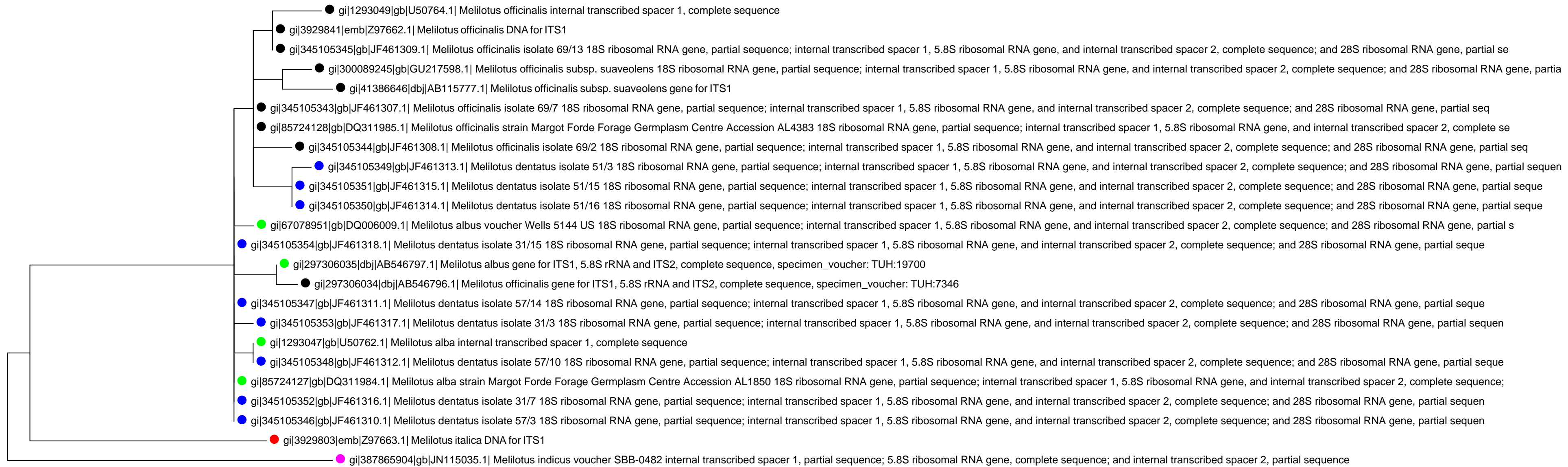

0.005

Supplement: S5 File — (PDF) [file pone.0145365.s005.pdf]

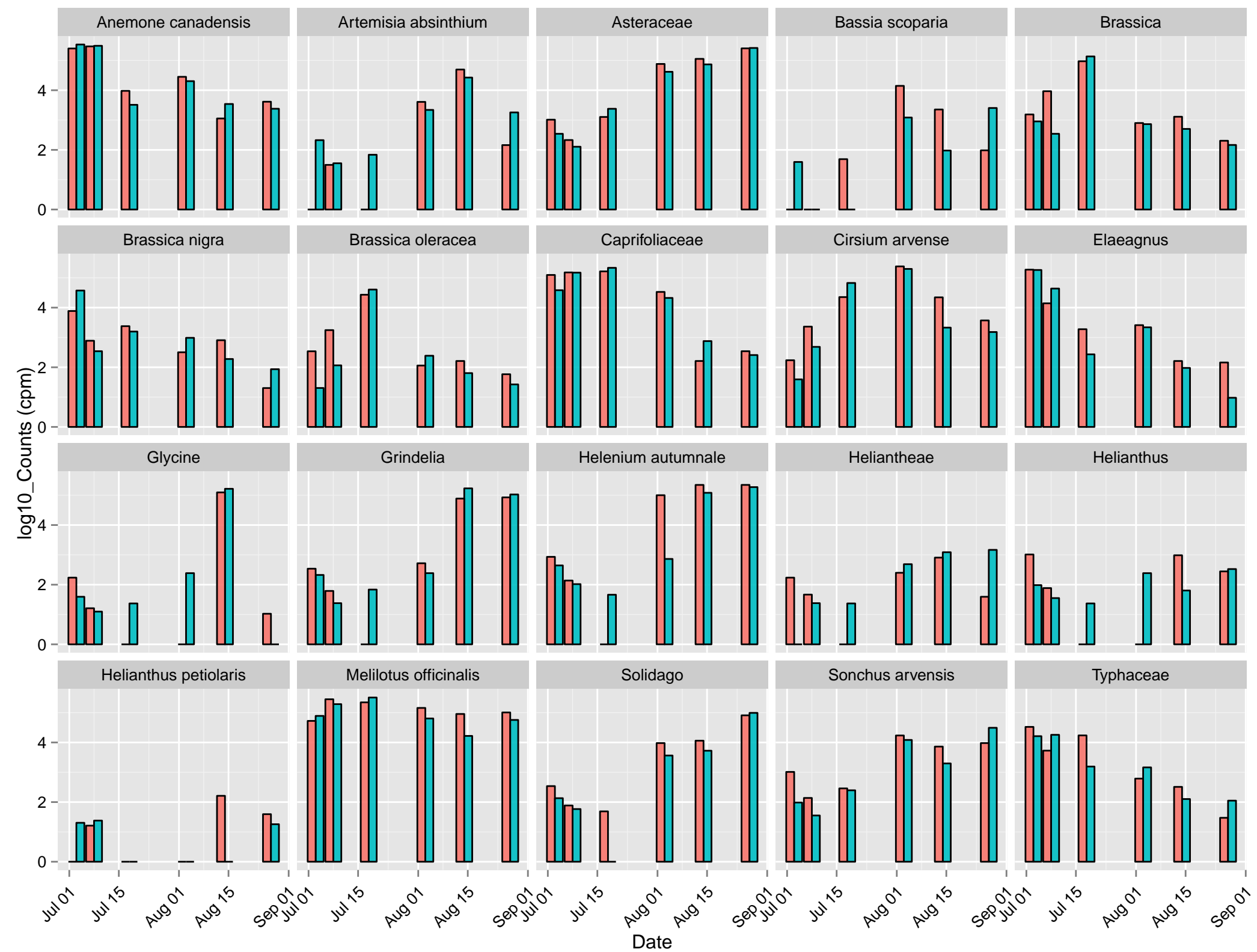

Supplement: S6 File — Combined OTU counts for the 20 most common plant assignments are represented, regardless of taxonomic level assignment. Vertical axis represents the log10 transformed counts per million mapped reads (cpm) for each taxon at each sampling point. (PDF) [file pone.0145365.s006.pdf]

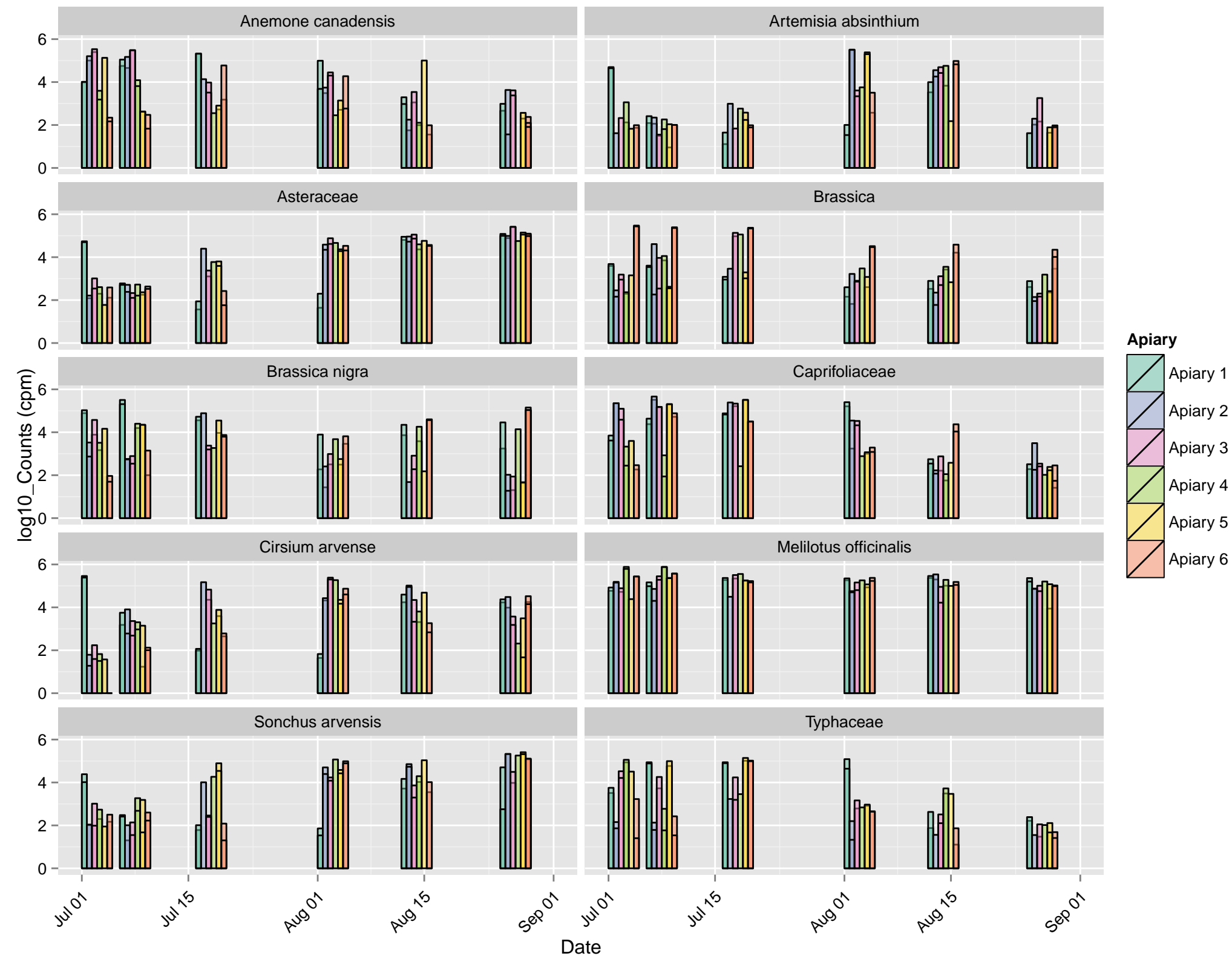

Supplement: S7 File — Combined OTU counts for the 10 most common plant assignments are represented, regardless of taxonomic level assignment. Vertical axis represents the log10 transformed counts per million mapped reads (cpm) for each taxon at each sampling point. Vertical bars are transparent to show data for multiple colonies sampled within the same apiary. (PDF) [file pone.0145365.s007.pdf]

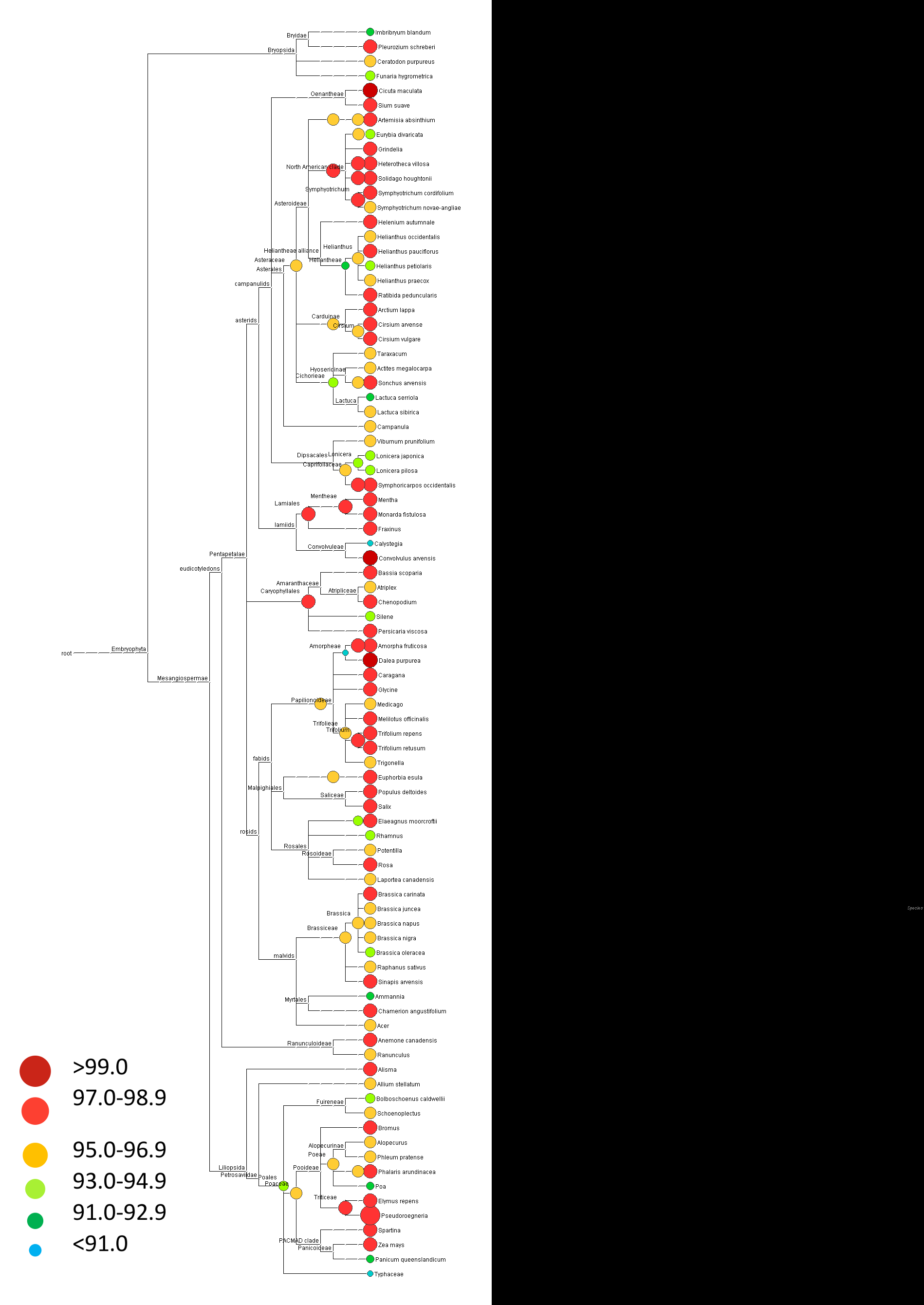

Supplement: S8 File — The phylogram was created with Megan v. 5.10.2 [52] using dummy counts appropriate to scale each node according to the legend. (TIF) [file pone.0145365.s008.tif]

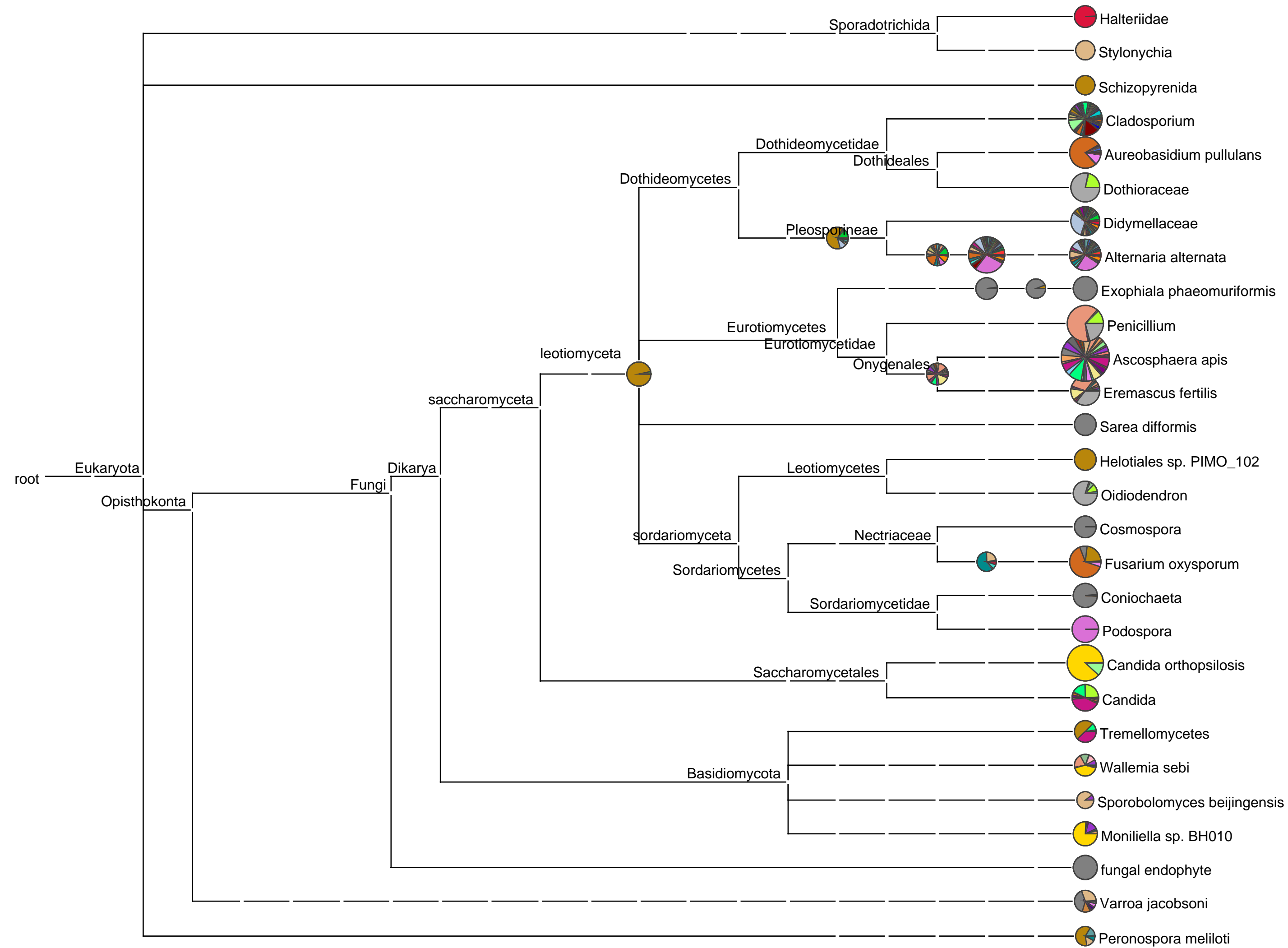

Supplement: S9 File — The area of each pie is proportional to log10(cpm), and each pollen sample is represented by an arbitrary color in order to illustrate that most taxa other than Ascosphaera apis were detected in few samples. (PDF) [file pone.0145365.s009.pdf]

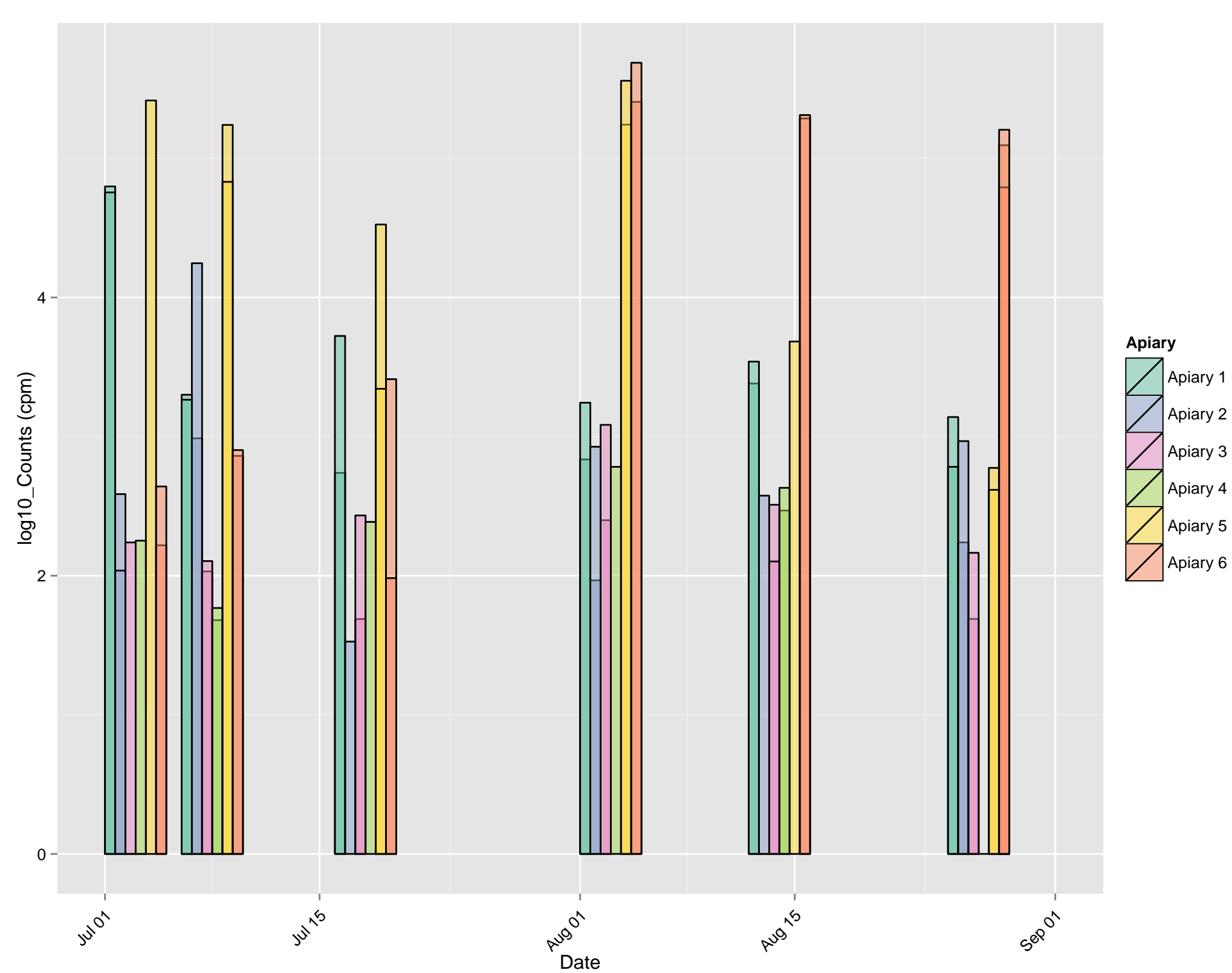

Supplement: S10 File — A background level of A. apis was found in all apiaries as well as apparent outbreaks in individual apiaries. (PDF) [file pone.0145365.s010.pdf]
